# Effectiveness and Quality of Life with Montelukast in Asthma – A double-blind randomized control trial

**DOI:** 10.12669/pjms.35.3.42

**Published:** 2019

**Authors:** Saifullah Baig, Rashid Ahmed Khan, Kamran Khan, Nadeem Rizvi

**Affiliations:** 1 *Dr. Mirza Saif Ullah Baig, MBBS, DTCD, MCPS, FCPS, Assistant Professor Pulmonology, Dow University of Health Sciences, Karachi, Pakistan*; 2 *Dr. Rashid Ahmed Khan, MBBS, DTCD, MCPS, FCPS. Head Department of Pulmonology. Liaquat University of Medical & Health Sciences, Jamshoro, Sindh, Pakistan*; 3 *Dr. Kamran Khan Sumalani, MBBS, DTCD, MCPS, Registrar, J.P.M.C, Karachi, Pakistan*; 4 *Dr. Nadeem Rizvi, MBBS, MCPS, MRCP, FRCP, Consultant Physician and Chest Specialist, Professor and Ex. Head of Chest Medicine, Jinnah Postgraduate Medical Center, Karachi, Pakistan*

**Keywords:** Anti-leukotrienes, leukotriene receptor antagonists, Montelukast, CysLT_1_ receptors, asthma, randomized controlled trial

## Abstract

**Objective:**

To determine the role of montelukast – a leukotriene receptor antagonist (LTRA) – in improving the quality of life (QOL) and asthma control of adult patients with mild to moderate persistent asthma.

**Methods:**

Randomized, double-blind, placebo-controlled, non-crossover trial was conducted from March 2017 till November 2018 in three hospitals of Karachi and Hyderabad. Adults of age 15 years or more with mild to moderate persistent asthma. Treatment group was administered tablet montelukast 10mg once daily; the other group was given a similar looking placebo; as an adjuvant to the current medication. QOL was assessed with Asthma Quality of Life Questionnaire – Standard (AQLQ-S) before and after the treatment. Asthma control was monitored via Asthma Control Test (ACT).

**Results:**

After 4 weeks, the mean ± SD of overall QOL on AQLQ-S improved from 3.74±0.88 to 5.06±0.89 for montelukast group and from 3.58±0.92 to 4.71±0.97 for placebo group (p=0.02). The improvement in sub-domains of symptoms, activity, and emotional functions was not significant; however, the sub-domain “environmental stimuli” significantly improved with 5.06±0.89 for montelukast group and 4.71±0.97 for placebo group (p=0.02). The mean ± SD of ACT, after four weeks, for montelukast group was 18.19±2.91 and for placebo group 17.28±3.36. Only on ACT, Montelukast did not show any statistically insignificant results.

**Conclusion:**

The role of montelukast in improving QOL of adult patients with mild to moderate persistent asthma is quite beneficial. It improves patient quality of life. It has the ease of once daily oral administration and also eradicates side effects associated with long-term adherence to steroids.

## INTRODUCTION

Asthma is the complex, chronic, immunologically mediated condition characterized by imbalance in the normal airway repair mechanism leading to airway inflammation, edema, obstruction, remodeling, and hyper-responsiveness. Acute asthmatic exacerbations are characterized by progressively worsening shortness of breath, chest tightness, cough, with or without wheezing. The underlying complex genetic actions and environmental triggers intricately interchange; making asthma a multifactorial syndrome of a broad array of variable phenotypes and manifestations.^1^

As the respiratory mucosa is exposed to an allergen, which maybe an environmental trigger or a microbe, a cascade of inflammatory response is initiated. This allergen is first endocytosed by the antigen presenting cells (APCs); which process it and present to naïve the T cells, which in turn, stimulate the respiratory epithelium to secrete inflammatory mediators such as thymic stromal lymphopoietin (TSLP) which recruit leukocytes and dendritic cells (DC). Under the influence of DCs, T cells are differentiated into T-helper 2 (Th2) and Th17 cells via stimulation of interleukin 4 (IL-4) and IL-13. IL-5, produced from Th2 cells, increases eosinophil levels which release inflammatory mediators. These chemicals, along with those released from T cells, macrophages, and neutrophils damage the airway, cause smooth muscle contraction, stimulate inflammatory pathways, and result in airway remodeling. Th2 cells also induce production of immunoglobulin E (IgE) antibody which binds to mast cells and basophils to release histamine, cysteinylleukotrienes (CysLTs) and other mediators.^2^

Leukotrienes activate by binding to G protein-coupled receptors present on the surfaces of airway cells. Once activated, CysLTs result in contraction of smooth muscle, increased vasculature leakage leading to edema, increased mucus secretion with decreased mucociliary clearance, and recruitment of leukocytes to the airway further augmenting the inflammation cascade.^2^,^3^ CysLTs results in airway remodelingby stimulating airway smooth muscle and epithelial muscle proliferation and by increasing collagen deposition. The majority of CysLTs mediated effects of asthma are regulated by CysLT_1_ as the effects are noted to be reversed with CysLT_1_antagonists.^4^

Even though the gold standard of long-term asthma care is inhaled corticosteroid (ICS); LTRAs have their advantage of easy oral dose, and reducing long-term side effects of ICS.^5^ Current Global Initiative for Asthma (GINA) guidelines recommend LTRAs as a monotherapy in mild persistent asthma, as an add-on or alternative to increasing dose of ICS or adding a long-acting β_2_-agonist.^6^ Montelukast inhibits the actions of leukotriene D4 (LTD4) by reversibly binding on the cysteinyl leukotriene receptor CysLT_1_ in the lungs and bronchial tubes. LTD4 is the most potent bronchoconstrictor and CysLT_1_ has chemo-attractive properties for many inflammatory cells. Hence, montelukast leukotriene induced decreases bronchoconstriction and resulting inflammation.^7^

Role of montelukast in controlling asthma and improving the quality of life (QOL) of asthmatic patients has been experimented.^8^-^10^ However, there are fewer double-blind randomized controlled trials that experimented the role of montelukast as an adjuvant therapy in controlling asthma and improving the QOL of asthmatic patients, especially from South Asian population. The aim of this study is to understand the role of montelukast (Aireez) in asthma control and QOL of asthmatic individuals.

## METHODS

It was a randomized, double-blind, placebo-controlled, non-crossover trial conducted for six months in the pulmonology department of a public tertiary care hospital in Karachi and Hyderabad, Pakistan. It was conducted from March 2017 till November 2018 in three hospitals of Karachi and Hyderabad – Taj Clinic, Jinnah Postgraduate Medical Center and Liaquat University of Medical and Health Sciences. Randomization was achieved by allocating all participant a random number sequence generated by a computer software. The study was approved by the institutional review board (No.: EC-107/CHEST/2017). The trial was also registered at www.clinicaltrials.gov. (Identifier: NCT03096327).

With 12% of incidence of Asthma in general population,^11^ the calculated sample size was 163 with power of the study at 80% and confidence interval of 95%. With consideration of dropout, 180 patients were invited to participate in the study. Any participant could withdraw from the clinical trial at any time after informing the Principal investigator. The participants had a clinical diagnosis of asthma for at least one year; were of age 15 years and more and of both genders. All participants were informed about the trial procession and provided with written informed consent.

However, participants who reported a previous incidence of adverse reaction to montelukast or any other leukotriene inhibitor; history of hyper-eosinophilic disorder other than atopic disease; treatment with montelukast within four weeks from randomization; and asthma exacerbation or treatment with prednisone/other systemic steroid within four weeks from randomization were excluded from the trial.

Of the 180 patients, 11 did not give consent, seven were excluded, and six dropped out. Therefore, 156 participants completed the trial. They were randomly divided into Placebo group (n=76) and treatment/montelukast group (n=80). Participants randomized to the treatment group received 10 mg montelukast once daily (OD). Patients in placebo group received identical looking tablet (placebo) produced by same manufacturer. Both products were packaged in identical strips identifiable only by the patient allocation number. All participants were asked to take one tablet each day. Acceptable compliance was defined as taking medication at least 21 days. The patients were instructed to leave the remaining tablets in their strip so that they were counted later. All participants were compliant.

This trial utilized two instruments; to assess the efficacy of treatment and to gauge the difference in quality of life in both the groups before and after receiving treatment for four weeks. Both instruments were self-administered and were translated in Urdu language for understanding and comprehension.

### Asthma Control Test (ACT)

In order to assess the efficacy of treatment, the participants of both groups had to fill a 5-question Asthma Control Test^12^ at the start of treatment and after four weeks. ACT gives a snapshot of asthma control over four weeks. Each question is marked on a Likert scale of 1-5 with 1 indicating the lowest control and 5 indicating the highest control. Mean score of less than 20 indicates uncontrolled asthma, mean score of 20-24 indicates reasonable control of asthma, and mean score of 25 indicates well controlled asthma.

### Asthma Quality of Life Questionnaire – Standard (AQLQ-S)

In order to assess the quality of life, the participants of both groups filled a 32-item Asthma Quality of Life Questionnaire – Standard^13^ at the start of treatment and after four weeks. It is a useful clinical measurement of quality of life of asthmatic patients over two weeks. It has four domains – (i) “symptoms” which includes 12 items and assesses the impact of asthma symptoms on the QOL; (ii) “activity” which includes 11 items and assesses the impact of asthma severity on activity limitation; (iii) ”emotions” which includes 5 items and assesses the impact asthma severity on emotional functionality; (iv) “environmental stimuli” which includes 4 items and assesses the impact of exposure to environmental stimuli on severity of asthma symptoms. It is graded on a 7-point Likert scale with higher AQLQ-S scores reporting better ambulatory functioning and QOL than those with lower AQLQ-S scores. The reliability score of AQLQ-S in this study was 0.87.

Data was collected using a study Performa which included patient characteristics such as patient allocation number, age, gender, asthma duration, clinical presentation and items of AQLQ-S and ACT. All participants completed AQLQ-S and ACT on Day 0 and Day 28 of the trial. Data was entered and analyzed using SPSS version 24. Mean and standard deviation (SD) was calculated for all numerical values such as age and scores of AQLQ-S and ACT. Frequencies were calculated for categorical data such as gender and clinical presentation. AQLQ-S Score for quality of life and ACT score asthma control was compared between treatment and placebo group using paired T-test. P values of less than 0.05 were considered to be statistically significant.

## RESULTS

One hundred and fifty-six participants who were randomly divided into placebo group (n=76) and treatment/montelukast group (n=80) completed the trial. Their Mean ± SD age was 36.7 ± 15.3 years and 66.7% of them were females (n=104). The demographics and clinical profile of the trial population is shown in Table-I.

**Table-I:** Demographics and Clinical feature of the patients (n=156).

Variable	Frequency (%)
Age in years (Mean ± SD)	36.7 ± 15.3
***Gender***	
Male	52 (33.3)
Female	104 (66.7)
***Clinical presentation***	
Cough	120 (76.9)
Wheezing	119 (76.3)
Chest tightness	91 (58.3)
Shortness of breath	140 (89.7)

***Abbreviations:*** SD, standard deviation.

At day 28, treatment group showed higher QOL than the placebo group overall as well as in all sub-domains of AQLQ-S. The difference was significant overall and for the sub-domain of environmental stimuli. The QOL score of the study sample at the start of treatment is shown in Table-II. In comparison, the improved QOL scores after one month of therapy are shown in Table-III.

**Table-II:** Comparison of Asthma Quality of Life Questionnaire score between montelukast and placebo group at Day 0 [Montelukast group n=80 (51.3), Placebo n= 76 (48.7)].

Aqlq-s score	Montelukast group Mean ± SD	Placebo group Mean ± SD	95% CI	P value*
Overall	3.74 ± 0.88	3.58 ± 0.92	- 0.12- 0.44	0.02*
Symptoms	3.97 ± 0.98	3.72 ± 1.10	-0.08- 0.57	0.13
Activity limitation	3.61 ± 0.98	3.44 ± 0.98	-0.13- 0.48	0.27
Emotional function	4.41 ± 1.15	4.36 ± 1.12	-0.31- 0.41	0.78
Environmental stimuli	2.60 ± 1.33	2.63 ± 1.25	-0.44-0.37	0.85

***Abbreviations:*** AQLQ-S, Asthma Quality of Life Questionnaire – Standard; SD, standard deviation; CI, confidence interval.

* Independent sample t test applied between groups; p value < 0.05 significant

**Table-III:** Comparison of Asthma Quality of Life Questionnaire score between montelukast and placebo group at Day 28 [Montelukast group n=80 (51.3), Placebo n= 76 (48.7)].

Aqlq-s score	Montelukast group Mean ± SD	Placebo group Mean ± SD	95% CI	P value*
Overall	5.06 ± 0.89	4.71 ± 0.97	0.05-0.64	0.02*
Symptoms	5.12 ± 0.86	4.83 ± 1.08	-0.01-0.60	0.06
Activity limitation	4.91 ± 0.89	4.65 ± 1.01	-0.46-0.55	0.09
Emotional function	5.45 ± 0.96	5.21 ± 1.15	-0.08-0.58	0.14
Environmental stimuli	4.09 ± 1.17	3.66 ± 1.20	0.06-0.81	0.02*

Abbreviations: AQLQ-S, Asthma Quality of Life Questionnaire – Standard; SD, standard deviation; CI, confidence interval.

* Independent sample t test applied between groups; p value < 0.05 significant

At day 28, the mean ± SD score of ACT questionnaire of the Montelukast group was 18.19 ± 2.91 and for placebo group it was 17.28 ± 3.36. Both indicated uncontrolled asthma. The difference was statistically insignificant.

## DISCUSSION

The results of this trial show that OD treatment with 10mg montelukast, in comparison to placebo, resulted in significant improvement in quality of life over a period of four weeks. There was overall significant QOL improvement as well as in the “environmental stimuli” sub-domain of AQLQ-S, in comparison to placebo. On the ACT scale, the patients showed improved response to “environmental stimuli” after therapy with montelukast; which corresponds to better asthma control. Overall asthma control might have also been seen in an improving trend, if the patients had been followed up for a longer duration.

Previous studies have established the role of LTRAs in managing asthma. In a randomized, double-blind, crossover trial by Lazarinis, and the effect of 20mg montelukast twice-daily for 5-7 days was compared with placebo in patients with mild asthma. Montelukast protected against LTE_4_-induced bronchoconstriction (p<0.001 vs placebo).^14^

Biernacki et al. concluded that the management of stable asthma with ICS alone is incomplete; and that adding LTRAs complement the effect of controlling inflammation; hence, significantly improving QOL.^10^ Similarly in another recent randomized, single-blind, placebo-controlled trial with acute asthmatic exacerbations, the treatment group received oral montelukast (10 mg, OD) for 2 weeks along with the standard therapy according to GINA guidelines. At week 2 and week 4, the montelukast group had higher peak expiratory flow rate (PEFR) (*P* < 0.0376 and *P* < 0.0003 respectively); and at week 4 the Montelukast group had higher forced expiratory volume in 1 second (*P* < 0.0033) as compared to the standard treatment alone.^15^ However, a local study did not show any benefit of adding montelukast to standard management of acute asthma exacerbations.^16^

The MONICA study also indicated that LTRAs as add-on therapy with ICS, long-acting β_2_-agonists (LABA) or both, improved both asthma control (on ACT scale; *p* < 0.0001) and asthma-related quality of life (on mini-AQLQ; *p* < 0.0001).^8^ Although Bozek et al. showed similar results in elderly asthmatic patients – lesser asthmatic exacerbations and lesser days with short acting beta-agonists (SABA)^9^; Columbo has produced contradictory results in elderly asthmatic patients. Though, their montelukast group had lesser daily symptoms scores and number of puffs of SABA after 8 weeks; the results were not statistically significant.^17^

In a meta-analysis conducted by Zhang et al., Montelukast significantly reduced the frequency of asthma exacerbations in contrast to placebo; its effect remained inferior to ICSs and ICS plus LABA as the first-line therapy and LABA as the add-on therapy.^18^

Pediatric as well as adult trials have been conducted to experiment the role of montelukast as an add-on agent for managing stable chronic asthma.^19^-^21^ In an adult-based, multicenter, phase IV trial that investigated the efficacy of montelukast 10 mg OD in adults with asthma and allergic rhinitis; the patients reported improved asthma symptoms. The use of other asthma medication was reduced and 92 patients intended to continue with montelukast. Overall QOL was “very good –good” in 85% patients.^22^

In another local study; the effects of monotherapy with oral montelukast were compared with ICS alone for 8 weeks. In montelukast group, PEFR rose steadily for first 4 weeks, but not in the ICS group. PEFR values at 1^st^ and 3^rd^ week favored Montelukast group (p <0.05); later it was statistically similar for both groups.^23^

The role of montelukast therapy in mild to moderate persistent asthma is very promising. It should be experimented as larger, multicenter trials for population-based results. The potential benefit of eliminating adverse effects of long-term steroids and also OD oral administration gives montelukast an unmatched precedence. It is one of the few double-blind trials from Pakistan. This trial puts forward the need to conduct larger population based double-blind trials which can follow the patients for longer durations to assess the long-term results of this therapy.

## CONCLUSION

Treatment with 10mg of montelukast in improving quality of life of adult patients with mild to moderate persistent asthma is quite beneficial as it has the ease of once daily oral administration and also reduce side effects associated with long-term adherence to steroids.
